# A twin and molecular genetics study of sleep paralysis and associated factors

**DOI:** 10.1111/jsr.12282

**Published:** 2015-02-09

**Authors:** Dan Denis, Christopher C. French, Richard Rowe, Helena M. S. Zavos, Patrick M. Nolan, Michael J. Parsons, Alice M. Gregory

**Affiliations:** ^1^ Department of Psychology, GoldsmithsUniversity of LondonLondonUK; ^2^ Department of PsychologyUniversity of SheffieldSheffieldUK; ^3^ Institute of PsychiatryKing's College LondonLondonUK; ^4^ Mammalian Genetics UnitMRC HarwellOxfordUK

**Keywords:** behavioural genetics, genetic association, Diurnal preference, sleep disruption

## Abstract

Sleep paralysis is a relatively common but under‐researched phenomenon. In this paper we examine prevalence in a UK sample and associations with candidate risk factors. This is the first study to investigate the heritability of sleep paralysis in a twin sample and to explore genetic associations between sleep paralysis and a number of circadian expressed single nucleotide polymorphisms. Analyses are based on data from the Genesis1219 twin/sibling study, a community sample of twins/siblings from England and Wales. In total, data from 862 participants aged 22–32 years (34% male) were used in the study. This sample consisted of monozygotic and dizygotic twins and siblings. It was found that self‐reports of general sleep quality, anxiety symptoms and exposure to threatening events were all associated independently with sleep paralysis. There was moderate genetic influence on sleep paralysis (53%). Polymorphisms in the PER2 gene were associated with sleep paralysis in additive and dominant models of inheritance—although significance was not reached once a Bonferroni correction was applied. It is concluded that factors associated with disrupted sleep cycles appear to be associated with sleep paralysis. In this sample of young adults, sleep paralysis was moderately heritable. Future work should examine specific polymorphisms associated with differences in circadian rhythms and sleep homeostasis further in association with sleep paralysis.

## Introduction

Sleep paralysis involves a period of inability to perform voluntary movements at either sleep onset or upon awakening (International Classification of Sleep Disorders, Third Edition, American Academy of Sleep Medicine, 2014). Sleep paralysis is often accompanied by a wide range of terrifying hallucinations (French and Santomauro, 2007). One systematic review suggested that 7.6% of individuals experienced sleep paralysis at least once in their lives, although individual estimates range widely between 2 and 60% (Sharpless and Barber, 2011). In the literature there is a distinction between sleep paralysis and isolated sleep paralysis (Sharpless *et al*., 2010). Sleep paralysis is a common symptom of narcolepsy. In those with narcolepsy, sleep paralysis may not occur independently of narcolepsy. When sleep paralysis occurs outside narcolepsy, it is termed ‘isolated sleep paralysis’ (Sharpless and Grom, 2014). As we were unable to rule out a narcolepsy diagnosis in our sample, we refrain from using the term isolated sleep paralysis.

The physiological mechanisms underlying sleep paralysis are unknown. Experimental evidence implicates disruption to the sleep cycle as a risk factor (Takeuchi *et al*., 2002). Furthermore, self‐reported sleep difficulties and consumption of alcohol, known to disrupt sleep, have both been associated with sleep paralysis (Munezawa *et al*., 2011). Epidemiological evidence also indicates that sleep paralysis is associated with anxiety disorders, self‐report levels of depression and exposure to trauma (Mellman *et al*., 2008; Ramsawh *et al*., 2009; Szklo‐Coxe *et al*., 2007).

Risk for sleep paralysis may, in part, be genetic. Currently, the role of genetic influences on sleep paralysis is unknown. Previous research has shown sleep paralysis to be familial. In one study of a single family, it was found that within the 64 members studied, 33 of them reported at least one sleep paralysis experience (Bell *et al*., 1986). In a second family study of frequent sleep paralysis sufferers, 19 of the 22 individuals investigated self‐reported sleep paralysis (Dahlitz and Parkes, 1993). While traditional family studies are able to demonstrate that traits run within families, they are not able to show whether similarity within family members comes from genetic or environmental factors (Plomin *et al*., 2013).

The present research uses a twin study to extend this literature in three ways. First, we examine concurrent associations between sleep paralysis and a range of potential risk factors. The measures are chosen based on previous literature on risk factors for sleep paralysis (outlined above). They are general sleep quality, anxiety symptoms, depressed mood, exposure to threatening events and weekly alcohol intake. We also examined smoking behaviour and caffeine intake as potential risk factors for sleep paralysis, on the basis that nicotine and caffeine have been linked to poor sleep quality (Karacan *et al*., 1976; Phillips and Danner, 1995). By looking at multiple variables simultaneously, we will be able to identify those associated independently with sleep paralysis.

Secondly, this is the first study to investigate genetic and environmental influences on sleep paralysis using a classical twin study design. Using this method, it is possible to estimate the extent of both genetic and environmental influences on sleep paralysis (Plomin *et al*., 2013). Thirdly, we investigate associations between sleep paralysis and 15 previously genotyped single nucleotide polymorphisms (SNPs). We focus on genes that have been investigated previously either in a sleep genomewide association study (GWAS) replication study or in a study investigating the role of genetic variation in circadian candidate genes involved in sleep homeostasis (see Parsons *et al*., 2013, 2014).

Table 1 summarizes the SNPs investigated here and their previous associations. The genes selected for inclusion have been linked to sleep/circadian cycles. Disruption to these cycles may be a risk for sleep paralysis, as deliberately induced sleep disruptions result in an increased frequency of sleep paralysis episodes (Takeuchi *et al*., 2002). A number of these SNPs have been linked to differences in diurnal preferences. Associations between sleep paralysis episodes and the time during sleep in which occur have been reported (Cheyne, 2002). Based on this, it may be that genetic variation in an individual's preference of timing of the sleep–wake cycle might be involved in sleep paralysis.

**Table 1:** Summary of investigated SNPs

Gene	SNP ID	Allele	Genotypic frequency			Related phenotype
			M/M	M/m	m/m	
PERI	rs2735611	T/C	419	129	14	Diurnal preference (Carpen *et al*., 2006)
PER2	rs934945	G/A	362	175	26	Diurnal preference (Carpen *et al*., 2006)
PER2	rs2304672	C/G	481	85	1	Diurnal preference/sleep duration/sleep quality (Carpen *et al*., 2006; Parsons *et al*., 2014)
PER3	rs2797687	C/A	361	164	19	Delayed sleep phase disorder (DSPD). Archer *et al*., 2003)
PER3	rs10462020	T/G	355	164	43	Diurnal preference (Parsons *et al*., 2014)
ABCC9	rs11046211	C/T	503	60	1	Sleep duration (Allebrandt *et al*., 2013)
ABCC9	rs11046209	A/T	505	60	1	Sleep duration (Allebrandt *et al*., 2013)
ABCC9	rs11046205	G/A	366	173	21	Sleep duration (Allebrandt *et al*., 2013)
CACNA1C	rs7304986	T/C	542	25	1	Sleep latency (Bryne *et al*., 2013)
CACNA1C	rs16929277	C/G	530	27	1	Sleep quality/sleep latency (Parsons *et al*., 2013)
CACNA1C	rs2302729	C/T	382	169	5	Sleep quality (Bryne *et al*., 2013)
ARNTL2	rs922270	T/C	414	143	12	Worse evening mood/diurnal preference (Parsons *et al*., 2014; Shi *et al*., 2010)
DBP	rs3848543	C/T	400	138	9	Worse evening mood (Shi *et al*., 2010)
CLOCK	rs2070062	T/G	304	214	48	Diurnal preference (Pedrazzoli *et al*., 2007)
GNβ3	rs5443	C/T	285	230	52	Sleep quality (Parsons *et al*., 2014)

## Method

### Participants

The present analyses used data from wave 5 of the G1219 longitudinal twin study (McAdams *et al*., 2013), the only wave at which sleep paralysis has been assessed. At wave 5, research assistants attempted to trace those who participated at wave 4 and their siblings (*n* = 1817). Following tracing, all 1817 participants were sent a 12‐page questionnaire booklet to complete. Overall, 883 booklets were completed. For full details on wave 5, see elsewhere (Barclay *et al*., submitted).

Zygosity was established through a parent‐reported questionnaire at waves 2 and 3, assessing physical similarity between twins (Cohen *et al*., 1975). When zygosity was available on only one or other wave, this rating was used. If there was disagreement between zygosity ratings at the two waves, DNA was obtained (*n* = 26 pairs) before final classifications were made. The total sample included in these analyses was 862 individuals. The mean age was 25.30 years [standard deviation (SD): 1.81, range: 22–32 years] and 66% of the sample were female. Individuals from 467 families: 118 monozygotic (MZ) twin pairs (105 complete), 222 dizygotic (DZ) twin pairs (182 complete) and 127 sibling pairs (91 complete). Zygosity type was unknown for 17 individuals.

Ethical approval for different stages of this study has been provided by the Research Ethics Committees of the Institute of Psychiatry, South London and Maudsley NHS Trust, and Goldsmiths, University of London.

### Measures

#### Sleep paralysis

Sleep paralysis was measured by a single item: ‘Sometimes, when falling asleep or waking up from sleep, I experience a brief period during which I feel I am unable to move, even though I think I am awake and conscious of my surroundings’. This item has been used in other work (Cheyne *et al*., 1999). Responses were on a seven‐point scale, ranging from never to several times a week. The label ‘sleep paralysis’ was not used, as labelling the experience can affect reported prevalence rate (Fukuda, 1993).

#### Sleep quality

Sleep quality was assessed using the Pittsburgh Sleep Quality Index (PSQI) (Buysse *et al*., 1989). This 18‐item questionnaire assesses seven components of sleep quality and disturbances (sleep duration, sleep disturbance, sleep latency, daytime dysfunction due to sleepiness, sleep efficiency, overall sleep quality and use of sleep medication). The global score has a theoretical range of 0–21, with a higher score indicating higher levels of sleep problems. The scale had a reliability of α = 0.71.

#### Anxiety symptoms

The Revised Symptoms of Anxiety Scale was used to measure anxiety in the present sample (Tom Willis, unpublished). This is a revised version of the Revised Child Anxiety and Depression Scale (Chorpita *et al*., 2000), adapted for use in longitudinal studies of anxiety in early adulthood. It is a 36‐item questionnaire yielding a total anxiety score. Participants are asked how often each item happens to them. Responses to each item are made on a four‐point scale (never, sometimes, often and always). The theoretical range of scores was 0–108, and a higher score indicates greater anxiety. The scale had a reliability of *α* = 0.94.

#### Depressed mood

The Moods and Feelings Questionnaire (MFQ) is a 13‐item questionnaire (Angold *et al*., 1995). Items ask how participants have been feeling or acting during the last 2 weeks. Responses to each item are on a three‐point scale (not true, sometimes and true). It has a theoretical range of 0–26, and yields a single global score measuring the core symptomatology of depression. A higher score indicates more depressive symptoms. The scale had a reliability of *α* = 0.90.

#### Threatening life events

Experience of threatening events during the last 12 months was measured using a list of 24 threatening life events (Brugha *et al*., 1985; Coddington and Humphrey, 1984). Examples of events include having been in hospital with a serious illness or injury or having had problems with the police or court appearance. A global score was calculated by tallying individual events experienced by each participant. A higher score indicated exposure to a greater number of potentially threatening events in the last 12 months. The theoretical range was 0–24 and had a reliability of *α* = 0.67.

#### Substance use

Measures of alcohol and smoking behaviour were based on previously used scales (Currie *et al*., 2008). Alcohol intake was assessed by four items: (1) ‘Do you drink?’; (2) ‘When you have an alcoholic drink, how many drinks do you have?’ [scale ranged from one to eight or more, with one alcoholic drink being ½ pint beer or lager/one glass of wine/one glass of spirits, or one ‘alcopop’ (a ready‐mixed drink that resembles a soft drink but contains alcohol—definition not given to participants)]; (3) ‘How often do you have an alcoholic drink’? (once or twice a year, once or twice in 6 months, once or twice a month, once or twice a week, three or four times a week, five or six times a week, almost every day); and (4) ‘During the last 30 days, how many times did you have five or more drinks on the same occasion?’ (four or more times, three times, twice, once, I have not had five or more drinks on the same occasion in the past month, I have never had five or more drinks on the same occasion). Using items 2–4, average alcohol intake in terms of units of alcohol consumed per week was calculated.

Smoking behaviour was assessed by three items: ‘Do you smoke?’, ‘How often do you smoke cigarettes?’ (every day, twice a week, once a week, once a fortnight and once a month) and ‘On the days that I do smoke, I smoke…’ (one‐five cigarettes, six–10 cigarettes, 11–15 cigarettes, 16–20 cigarettes, more than 20 cigarettes). The latter two questions were then used to calculate the number of cigarettes smoked per week.

The number of caffeinated drinks consumed per day for the last month was assessed (Kendler and Prescott, 1999). The drinks considered were: freshly brewed coffee (one shot espresso = one cup), instant coffee, caffeinated tea, and caffeinated soft drinks, and the numbers of each drink type consumed ranged from 0 to 8 or more. The number of each drink type consumed was recoded to reflect the amount of caffeine present in each type of drink. By adding together the score for each drink type, total caffeine intake was calculated.

### Genetic analyses

#### The classical twin method

Twin studies compare within‐pair similarity for groups of MZ twins, who are genetically identical, and DZ twins, who share on average half their segregating genes. This information can be used to estimate additive genetic (A), dominant genetic (D), shared environmental (C) and non‐shared environmental (E) influences on a trait (Plomin *et al*., 2013). Additive genetic influences refer to individual differences in a phenotype influenced by the additive effect of independent alleles. Dominant genetic influences are when the influence of one allele interacts with another at a locus to influence behaviour. Shared environmental influences make individuals within a family more similar, and non‐shared environmental influences make individuals within a family less similar, and also account for error. It is not possible to estimate dominant genetic and shared environmental influences in the same model, so either an ACE or ADE model can be fitted to the data. More detailed coverage of the classical twin design is available elsewhere (Plomin *et al*., 2013).

#### DNA extraction and genotyping

Cheek swab kits were posted to participants in order to collect DNA, primarily during wave 4; see elsewhere for a detailed description of DNA extractions (Barclay *et al*., 2011). Fifteen SNPs were genotyped (see Table 1). The SNPs rs11046209 and rs11046211 as well as rs16929277 and rs7304986 were proxies for each other and were included as technical replicates. All the genotyping assays were performed by KBioscience using KASPar chemistry; for more details see elsewhere (LGC Genomics, 2014). Blind duplicates and Hardy–Weinberg equilibrium (HWE) tests were used as quality control tests (Turner *et al*., 2011). Linkage disequilibrium (LD) and HWE were calculated using the Haploview program (Barrett *et al*., 2005). Both the proxy pairs were in complete LD with each other (*R* ^2^ = 1), so one SNP from each pair was included in the analysis (rs11046209 and rs16929277, respectively). The SNPs rs10462020 (*χ* ^2^ = 13.74, *P* = 0.0004) and rs2302729 (*χ* ^2^ = 8.73, *P* = 0.0025) failed to reach HWE and were therefore dropped from the analyses. All other SNPs were in HWE at the threshold of *P *<* *0.05. The SNPs rs934945 and rs2304672 occurred in the same gene (PER2), and were not in LD with each other (*χ* ^2^ = 6.82, *P* = 0.004). The SNPs rs11046209 and rs11046205 also occurred in the same gene (ABCC9), and were not in LD with each other (*χ* ^2^ = 25.55, *P *<* *0.001).

### Statistical analyses

The sleep paralysis measure was heavily positively skewed (skew = 1.93). As transforming the variable was not able to improve the distribution sufficiently, the variable was dichotomized into sleep paralysis absent = 0 and sleep paralysis present = 1. Participants who had experienced sleep paralysis at least once or more in their lives were categorized as sleep paralysis present. All analyses were performed on the dichotomized variable. Age and sex were controlled in all analyses. Phenotypic and molecular genetic analyses were carried out using Stata 9 (StataCorp, 2005). The Stata command ‘cluster’ was used in order for the tests to be robust against the non‐independence of observations found in the twin sample. Logistic regression was used to explore associations between variables, and all continuous predictors were standardized. Cases with missing data were removed prior to analysis. Twin analysis was carried out using OpenMx (Boker *et al*., 2011). The sleep paralysis variable was analysed using a univariate threshold liability model. Logistic regression analyses were conducted to model the main effect of SNPs on sleep paralysis. Three non‐independent models of inheritance were investigated: additive, dominant and recessive. The *P*‐value was adjusted by using the total number of SNPs investigated for sleep paralysis, thus the corrected *P*‐values are *P* = 0.05/11 = 0.0045. We did not apply corrections for multiple testing for the number of inheritance models that we ran (additive, dominant, and recessive), as these tests were not independent of each other.

## Results

### Prevalence of SP

**Figure 1:**
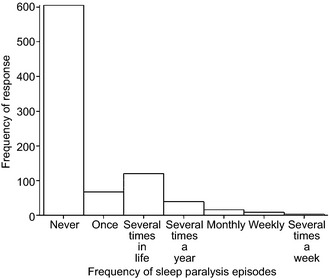
Histogram showing the distribution of sleep paralysis frequency in the current sample.

Descriptive statistics for all variables are provided in Table 2. Of the sample, 29.7% reported experiencing sleep paralysis at least once in their lives. A smaller percentage, 7.9%, reported experiencing sleep paralysis several times a year. The distribution of sleep paralysis scores is displayed in Fig. 1. Using the dichotomous sleep paralysis measure there were no sex differences, but there was a significant relationship between sleep paralysis and age (see Table 3).

**Table 2:** Descriptive statistics and correlations matrix for all variables assessed in the study

Variable	Mean (SD)	Sleep paralysis	Sleep quality	Anxiety	Depression	Threatening events	Alcohol	Smoking behaviour	Caffeine
Sleep paralysis	1.67 (1.16)	1.00							
Sleep quality	5.38 (5.30)	0.24***	1.00						
Anxiety symptoms	22.13 (14.81)	0.28***	0.35***	1.00					
Depressed mood	5.31 (5.30)	0.28***	0.45***	0.64***	1.00				
Threatening events	1.92 (2.13)	0.21***	0.26***	0.17***	0.30***	1.00			
Alcohol	5.91 (7.38)	0.08*	−0.02	−0.08*	−0.01	0.09*	1.00		
Smoking behaviour	7.00 (22.20)	0.16***	0.11**	0.10**	0.18***	0.25*	0.20***	1.00	
Caffeine	7.71 (8.00)	0.04	0.09*	0.07*	0.12**	0.10**	0.18***	0.18***	1.00

* = p < .05, ** = p < .01, *** = p < .001

**Table 3:** Associations of standardised predictor scores with sleep paralysis

Variable	Single predictor models		Multiple predictor model	
	OR (CI)	SE	OR (CI)	SE
Sleep quality	1.68*** (1.42–1.98)	0.14	1.28* (1.05–1.56)	0.13
Anxiety symptoms	1.76*** (1.51–2.05)	0.14	1.39** (1.13–1.71)	0.15
Depressed mood	1 82*** (1.54–2.14)	0.15	1.23 (0.99–1.53)	0.14
Threatening events	1.53*** (1.30–1.79)	0.12	1.29** (1.08–1.54)	0.11
Alcohol	1.16* (1.00–1.33)	0.08	1.12 (0.95–1.32)	0.10
Smoking behaviour	1.15 (0.99–1.33)	0.09		
Caffeine	1.08 (0.93–1.25)	0.08		
Age	0.88** (0.81–0.96)	0.04		
Sex	1.09 (0.78–1.52)	0.17		

OR, odds ratio; CI, confidence interval; SE, standard error.

* = p < .05, ** = p < .01, *** = p < .001

### Phenotypic analysis

The correlation matrix for all variables is displayed in Table 2. Results of the logistic regression models are displayed in Table 3. In age‐ and sex‐controlled single predictor models, all the variables except for smoking behaviour and caffeine intake were associated significantly with sleep paralysis. All the variables associated significantly with sleep paralysis in single predictor models (sleep quality, anxiety symptoms, depressed mood, exposure to threatening events and alcohol intake) were entered into the multiple predictor model to test associations simultaneously. In this model, sleep quality, anxiety symptoms and exposure to threatening events were associated significantly with sleep paralysis.

### Univariate twin model

Tetrachoric twin correlations for sleep paralysis were higher for MZ (*rt* = 0.56) than DZ (*rt* = −0.004) twins. Furthermore, MZ correlations were higher than sibling correlations (*rt* = 0.15). Fisher's *r–z* transform was used to establish whether the differences between the magnitude of the correlations were significant (Kenny, 1987). The MZ twin correlations were significantly higher than the DZ twins (*z* = 13.67, *P *<* *0.001). MZ correlations were also significantly higher than siblings (*z* = 9.66, *P *<* *0.001).

This pattern of results suggests a heritable component. Dominant genetic influence was implied, as the MZ correlations were more than double the DZ correlation (Plomin *et al*., 2013). The fact that the DZ correlation was near zero suggests that no additive genetic or shared environmental components were involved in explaining variation in sleep paralysis.

In line with the twin correlations, an ADE threshold liability model provided a good fit. There was a non‐significant deterioration in fit when compared to the saturated model (−2LL = 1529.4, df = 1250, *P* = 0.32). The model estimated: A = 0.00 [95% confidence interval (CI): 0.00–0.47]; D = 0.53 (95% CI: 0.00–0.70); E = 0.47 (95% CI: 0.30–0.68). The fit of a submodel was then tested by dropping the A and D components, thus testing the importance of genetic effects in explaining the sleep paralysis phenotype. This model showed a significant deterioration in fit, compared to the full ADE model (−2LL = 1551.5, df = 1252, *P *<* *0.001), highlighting the importance of genetic influences on sleep paralysis.

### SNP associations with sleep paralysis

The genotypic frequencies for the investigated SNPs are presented in Table 1. The mean sleep paralysis scores by genotype are displayed in Table 4. Odds ratios (ORs) for all SNPs are displayed in Table 5.

**Table 4:** Means and standard deviations of sleep paralysis by genotype

Gene	SNP	Genotype	Total Mean (SD)	Males Mean (SD)	Females Mean (SD)
PER1	rs2735611	TT	1.68 (1.21)	1.62 (1.13)	1.70 (1.25)
		TC	1.47 (0.93)	1.45 (0.96)	1.48 (1.25)
		CC	1.64 (0.93)	1.66 (1.15)	1.63 (0.92)
PER2	rs934945	GG	1.62 (1.11)	1.64 (1.15)	1.60 (1.08)
		GA	1.70 (1.25)	1.43 (0.88)	1.84 (1.38)
		AA	1.43 (0.95)	1.73 (1.27)	1.20 (0.56)
PER2	rs2304672	CC	1.57 (1.09)	1.53 (0.97)	1.60 (1.16)
		CG	1.85 (1.28)	1.81 (1.60)	1.86 (1.22)
		GG	5.00 (−)	5.00 (−)	–
PER3	rs2797687	CC	1.63 (1.15)	1.61 (1.10)	1.64 (1.17)
		CA	1.55 (1.10)	1.47 (0.96)	1.60 (1.18)
		AA	1.95 (1.08)	1.66 (1.03)	2.08 (1.12)
ABCC9	rs11046209	AA	1.60 (1.10)	1.57 (1.06)	1.63 (1.13)
		AT	1.80 (1.36)	1.50 (1.10)	1.97 (1.48)
		TT	1.00 (−)	–	1.00 (−)
ABCC9	rs11046205	GG	1.61 (1.15)	1.57 (1.10)	1.64 (1.18)
		GA	1.65 (1.13)	1.65 (1.08)	1.65 (1.16)
		AA	1.66 (1.24)	1.38 (1.06)	1.85 (1.34)
CACNA1C	rs16929277	CC	1.62 (1.14)	1.59 (1.08)	1.64 (1.17)
		CG	1.60 (1.15)	1.27 (0.80)	2.00 (1.41)
		GG	1.00 (−)	–	1.00 (−)
ARNTL2	rs922270	TT	1.65 (1.16)	1.56 (1.03)	1.71 (1.24)
		TC	1.54 (1.08)	1.62 (1.25)	1.51 (1.01)
		CC	1.75 (1.22)	2.50 (2.12)	1.60 (1.07)
CLOCK	rs2070062	TT	1.61 (1.16)	1.41 (0.95)	1.71 (1.25)
		TG	1.61 (1.07)	1.68 (1.09)	1.57 (1.07)
		GG	1.80 (1.21)	2.00 (1.45)	1.64 (1.13)
DBP	rs3848543	CC	1.64 (1.15)	1.57 (1.07)	1.67 (1.19)
		CT	1.51 (1.03)	1.53 (1.02)	1.51 (1.03)
		TT	2.11 (1.62)	1.67 (0.58)	2.33 (1.97)
GNβ3	rs5443	CC	1.62 (1.14)	1.55 (1.13)	1.65 (1.14)
		CT	1.62 (1.23)	1.58 (1.02)	1.64 (1.20)
		TT	1.70 (1.23)	1.60 (1.06)	1.74 (1.30)

SD, standard deviation.

**Table 5:** Odds ratios from logistic regression analysis for main effects of genotype on sleep paralysis measure

Gene	SNP	Model	Odds ratio (SE)
PER1	rs2735611	Additive	0.87 (0.17)
		Recessive	1.43 (0.76)
		Dominant	0.02 (0.47)
PER2	rs934945	Additive	0.97 (0.15)
		Recessive	0.74 (0.34)
		Dominant	1.02 (0.19)
PER2	rs2304672	Additive	1.88 (0.45)**
		Recessive	–
		Dominant	1.84 (0.45)*
PER3	rs2797687	Additive	1.03 (0.18)
		Recessive	2.35 (1.04)
		Dominant	0.90 (0.18)
ABCC9	rs11046205	Additive	2.06 (0.18)
		Recessive	0.78 (0.46)
		Dominant	1.13 (0.22)
ABCC9	rs11046209	Additive	1.13 (0.33)
		Recessive	–
		Dominant	1.17 (0.35)
CACNA1C	rs16929277	Additive	0.84 (0.35)
		Recessive	–
		Dominant	0.87 (0.38)
ARNTL2	rs922270	Additive	0.84 (0.16)
		Recessive	1.27 (0.66)
		Dominant	0.78 (0.16)
CLOCK	rs2070062	Additive	1.13 (0.16)
		Recessive	1.38 (0.40)
		Dominant	1.13 (0.21)
DBP	rs3848543	Additive	0.97 (0.19)
		Recessive	3.18 (1.89)
		Dominant	0.86 (0.18)
GNβ3	rs5443	Additive	1.05 (0.15)
		Recessive	1.06 (0.33)
		Dominant	1.06 (0.20)

SE, standard error. * = p < .05, ** = p < .01

Using a standard approach to significance testing, there was a significant association between the PER2 SNP rs2304672 and sleep paralysis using an additive model of inheritance (*P* = 0.008) and a dominant model of inheritance (*P* = 0.04). The relationship remained significant in the additive model when a single individual with the rare GG genotype was removed from the data set (*P* = 0.02). The other PER2 SNP investigated (rs934945) was not in LD with this SNP, so no association between the two SNPs was expected. None of the associations remained significant once the Bonferoni correction was applied (*P* = 0.0045).

## Discussion

In this large‐scale community sample, approximately one‐third of participants reported experiencing sleep paralysis at least once in their lives. This is, to our knowledge, the first prevalence estimate of sleep paralysis in UK residents, and is higher than a recent review paper suggesting that sleep paralysis occurs in only 7.6% of the general population (Sharpless and Barber, 2011). However, we found a similar prevalence estimate to other research using the same item to measure sleep paralysis (Cheyne *et al*., 1999).

The phenotypic analyses showed that sleep quality, anxiety and exposure to threatening events were related independently to sleep paralysis. Smoking behaviour and caffeine intake were not associated significantly with sleep paralysis. Alcohol intake and depressed mood were associated significantly with sleep paralysis when considered in isolation, but not when considered with multiple variables, suggesting that they are not unique predictors of sleep paralysis.

These findings are in line with previous research. Sleep paralysis has been shown to be associated with higher levels of self‐reported stress and trauma (Mellman *et al*., 2008). Similarly, our findings support previous research linking anxiety to sleep paralysis (Ramsawh *et al*., 2009). Our results did not find any independent association between sleep paralysis and depressed mood, contrary to other research (Szklo‐Coxe *et al*., 2007). This may be due to the higher number of predictor variables included in this study that showed an independent relationship with sleep paralysis.

This is the first twin study investigating the aetiology of sleep paralysis. Removing the genetic components from the model led to a significant deterioration in model fit, suggesting that genetic effects are important in explaining individual differences for experiencing sleep paralysis.

Based on the heritability indicated by the twin analyses, we examined the role of specific genes for which variation had been measured in the G1219 sample. This is the first study to our knowledge to look at associations between gene polymorphisms and sleep paralysis. We found that variation in the PER2 gene was associated with sleep paralysis in additive and dominant models, although not when Bonferroni correction was applied. The PER2 gene is one of several ‘clock’ genes that govern circadian rhythms, and act as regulators of the molecular clock. Variations in this gene in humans have also been linked to differences in diurnal preference (Parsons *et al*., 2014). Here we found that variation in this gene is also associated with sleep paralysis, although not when a Bonferroni correction was applied. If a significant association is found in future studies, this would suggest that variation in sleep and circadian genes partially underlie the inheritance seen for sleep paralysis in the twin analysis.

### Limitations

The results need to be considered alongside limitations. First, when considering the phenotypic analyses it is important to note that the data were cross‐sectional, preventing conclusions about the direction of effects. It is therefore possible that sleep paralysis could have led to other symptoms (e.g. anxiety) or that a third phenotype could have influenced both sleep paralysis and the associated factor. It should also be considered that all the data were based on self‐report measures.

In the twin analysis, given the small sample size relative to many behavioural genetic studies and the use of a dichotomous sleep paralysis variable, it would be unwise to distinguish between additive and dominant genetic effects. Instead, it may be more sensible to interpret this finding in terms of broad sense heritability. As well as the small sample size, the relatively young age of participants in the sample (range: 22–32) may make it difficult to generalize the findings to other age groups.

In the molecular genetic analysis, selection of candidate genes was exploratory, meaning that a large Bonferroni correction was applied to control for the multiple comparisons, increasing the chances of false negatives occurring. However, this approach is justified, because there is no previous research investigating genes associated with sleep paralysis. Future work should aim to replicate and extend these findings in a larger sample, and using alternative approaches such as GWAS.

## Conclusions

In conclusion, anxiety symptoms, exposure to threatening events and general sleep quality are all independent predictors of sleep paralysis. Sleep paralysis appears to have a genetic component, and future work should attempt to identify candidate genes which may be involved. While replication of these findings is necessary, it is hoped that this preliminary work will encourage further investigation of the genetics of sleep paralysis.

## Conflict of Interest

The authors declare no conflicts of interest.

## Author Contributions

DD, CCF and AMG designed the study. DD collected the data. PMN funded the genotyping. DD, RR, HMSZ, MJP, and AMG analysed the data. All authors were involved in the preparation of the manuscript.

## References

[jsr12282-bib-0001] Allebrandt, K. V. , Amin, N. , Müller‐Myhsok, B. *et al* A K(ATP) channel gene effect on sleep duration: from genome‐wide association studies to function in *Drosophila* . Mol. Psychiatry, 2013, 18: 122–132. 22105623 10.1038/mp.2011.142

[jsr12282-bib-0002] American Academy of Sleep Medicine . The International Classification of Sleep Disorders, 3rd edn American Academy of Sleep Medicine, Darien, IL, 2014.

[jsr12282-bib-0003] Angold, A. , Costello, E. J. , Messer, S. C. , Pickles, A. , Winder, F. and Silver, D. The development of a short questionnaire for use in epidemiological studies of depression in children and adolescents. Int. J. Methods Psychiatr. Res., 1995, 5: 237–249.

[jsr12282-bib-0004] Archer, S. N. , Robilliard, D. L. , Skene, D. J. *et al* A length polymorphism in the circadian clock gene Per3 is linked to delayed sleep phase syndrome and extreme diurnal preference. Sleep, 2003, 26: 413–415. 12841365 10.1093/sleep/26.4.413

[jsr12282-bib-0005] Barclay, N. L. , Eley, T. C. , Mill, J. *et al* Sleep quality and diurnal preference in a sample of young adults: associations with 5HTTLPR, PER3, and CLOCK 3111. Am. J. Med. Genet. B Neuropsychiatr. Genet., 2011, 156: 681–690. 21714069 10.1002/ajmg.b.31210

[jsr12282-bib-0007] Barrett, J. C. , Fry, B. , Maller, J. and Daly, M. J. Haploview: analysis and visualisation of LD and haplotype maps. Bioinformatics, 2005, 21: 263–265. 15297300 10.1093/bioinformatics/bth457

[jsr12282-bib-0008] Bell, C. C. , Dixie‐Bell, D. D. and Thompson, B. Further studies on the prevalence of isolated sleep paralysis in black subjects. JAMA, 1986, 78: 649–659. PMC2571385 3746934

[jsr12282-bib-0009] Boker, S. , Neale, M. , Maes, H. *et al* OpenMx: an open source extended structural equation modelling framework. Psychometrika, 2011, 76: 306–317. 23258944 10.1007/s11336-010-9200-6 PMC3525063

[jsr12282-bib-0010] Brugha, T. , Bebbington, P. , Tennant, C. and Hurry, J. The list of threatening experiences—a subset of 12 life events categories with considerable long‐term contextual threat. Psychol. Med., 1985, 15: 189–194. 3991833 10.1017/s003329170002105x

[jsr12282-bib-0011] Bryne, E. M. , Gehrman, P. R. , Medland, S. E. *et al* A genome‐wide association study of sleep habits and insomnia. Am. J. Med. Genet. B Neuropsychiatr. Genet., 2013, 162: 439–451. 10.1002/ajmg.b.32168 PMC4083458 23728906

[jsr12282-bib-0012] Buysse, D. J. , Reynolds, C. , Monk, T. , Berman, S. and Kupfer, D. The Pittsburgh Sleep Quality Index (PSQI): a new instrument for psychiatric practice and research. Psychiatry Res., 1989, 28: 193–213. 2748771 10.1016/0165-1781(89)90047-4

[jsr12282-bib-0013] Carpen, J. D. , von Schantz, M. , Smits, M. , Skene, D. J. and Archer, S. N. A silent polymorphism in the PER1 gene associates with extreme diurnal preference in humans. J. Hum. Genet., 2006, 51: 1122–1125. 17051316 10.1007/s10038-006-0060-y

[jsr12282-bib-0014] Cheyne, J. A. Situational factors affecting sleep paralysis and associated hallucinations: position and timing effects. J. Sleep Res., 2002, 11: 169–177. 12028482 10.1046/j.1365-2869.2002.00297.x

[jsr12282-bib-0015] Cheyne, J. A. , Newby‐Clark, I. R. and Rueffer, S. D. Relations among hypnogogic and hypnopompic experiences associated with sleep paralysis. J. Sleep Res., 1999, 8: 313–317. 10646172 10.1046/j.1365-2869.1999.00165.x

[jsr12282-bib-0016] Chorpita, B. , Yim, L. , Moffitt, C. , Umemoto, L. and Francis, S. Assessment of symptoms of DSM‐IV anxiety and depression in children: a revised child anxiety and depression scale. Behav. Res. Ther., 2000, 38: 835–855. 10937431 10.1016/s0005-7967(99)00130-8

[jsr12282-bib-0017] Coddington, R. D. and Humphrey, J. H. eds. Measuring the stressfulness of a child's environment In: Coddington R. D. and Humphrey J. H. (Eds) Stress in Childhood. AMS Press Inc., New York, 1984: 97–126.

[jsr12282-bib-0018] Cohen, D. J. , Dibble, E. , Grawe, J. M. and Pollin, W. Reliably separating identical from fraternal twins. Arch. Gen. Psychiatry, 1975, 32: 1371–1475. 1239251 10.1001/archpsyc.1975.01760290039004

[jsr12282-bib-0019] Currie, C. , Samdal, O. , Boyce, W. and Smith, R. Researching health inequalities in adolescents: the development of the health behaviour in school‐aged children (HBSC) family affluence scale. Soc. Sci. Med., 2008, 66: 1439–1436. 10.1016/j.socscimed.2007.11.024 18179852

[jsr12282-bib-0020] Dahlitz, M. and Parkes, J. D. Sleep paralysis. Lancet, 1993, 341: 406–407. 8094172 10.1016/0140-6736(93)92992-3

[jsr12282-bib-0021] French, C. C. and Santomauro, J. Something wicked this way comes: causes and interpretations of sleep paralysis In: Della Sala S. (Ed.) Tall Tales About the Mind and Brain: Separating Fact From Fiction. Oxford University Press, Oxford, 2007; 380–398.

[jsr12282-bib-0022] Fukuda, K. One explanatory basis for the discrepancy of reported prevalence of sleep paralysis among healthy respondents. Percept. Mot. Skills, 1993, 77: 803–807. 8284156 10.2466/pms.1993.77.3.803

[jsr12282-bib-0023] Karacan, I. , Thornby, J. I. , Anch, M. , Booth, G. H. , Williams, R. L. and Salis, P. J. Dose‐related sleep disturbances induced by coffee and caffeine. Clin. Pharmacol. Ther., 1976, 20: 682–689. 186223 10.1002/cpt1976206682

[jsr12282-bib-0024] Kendler, K. and Prescott, C. Caffeine intake, tolerance, and withdrawal in women. A population‐based twin study. Am. J. Psychiatry, 1999, 156: 223–228. 9989558 10.1176/ajp.156.2.223

[jsr12282-bib-0025] Kenny, D. A. Statistics for Social and Behavioural Sciences. Little, Brown and Company, Canada, 1987; 274–277.

[jsr12282-bib-0026] LGC Genomics . 2014 KASP overview [accessed 9 September 2014] (about 2 screens). Available at: http://www.lgcgenomics.com/genotyping/kasp-genotyping-chemistry/kasp-overview/?data=genotyping/kasp-genotyping-reagents/kasp-overview

[jsr12282-bib-0027] McAdams, T. A. , Gregory, A. M. , Rowe, R. *et al* The Genesis 12‐19 (G1219) study: a twin and sibling study of gene–environment interplay and adolescent development in the UK. Twin Res. Hum. Genet., 2013, 16: 134–143. 23394190 10.1017/thg.2012.83

[jsr12282-bib-0028] Mellman, T. A. , Aigbogun, N. , Graves, R. E. , Lawson, W. B. and Alim, T. N. Sleep paralysis and trauma, psychiatric symptoms and disorders in an adult African American population attending primary medical care. Depress. Anxiety, 2008, 5: 435–440. 17607754 10.1002/da.20311

[jsr12282-bib-0029] Munezawa, T. , Kaneita, Y. , Osaki, Y. *et al* Nightmare and sleep paralysis among Japanese adolescents: a nationwide representative survey. Sleep Med., 2011, 12: 56–64. 20920888 10.1016/j.sleep.2010.04.015

[jsr12282-bib-0030] Parsons, M. J. , Lester, K. J. , Barclay, N. L. , Nolan, P. M. , Eley, T. C. and Gregory, A. M. Replication of genome‐wide association studies (GWAS) loci for sleep in the British G1219 cohort. Am. J. Med. Genet. B Neuropsychiatr. Genet., 2013, 162: 331–438. 10.1002/ajmg.b.32106 23780892

[jsr12282-bib-0031] Parsons, M. J. , Lester, K. J. , Barclay, N. L. *et al* Polymorphisms in the circadian expressed genes PER3 and ARNTL2 are associated with diurnal preference and GNβ3 with sleep measures. J. Sleep Res., 2014, 23: 1–10. 10.1111/jsr.12144 PMC4320759 24635757

[jsr12282-bib-0032] Pedrazzoli, M. , Louzada, F. M. , Pereira, D. S. *et al* Clock polymorphisms and circadian rhythms phenotypes in a sample of the Brazilian population. Chronobiol. Int., 2007, 24: 1–8. 17364575 10.1080/07420520601139789

[jsr12282-bib-0033] Phillips, B. A. and Danner, F. J. Cigarette smoking and sleep disturbance. Arch. Intern. Med., 1995, 155: 734–737. 7695462

[jsr12282-bib-0034] Plomin R., DeFries J. C., Knopik V. S. and Neiderhiser J. M. (Eds) Behavioral Genetics, 6th edn Worth Publishers, New York, 2013.

[jsr12282-bib-0035] Ramsawh, H. J. , Stein, M. B. , Belik, S. L. , Jacobi, F. and Sareen, J. Relationship of anxiety disorders, sleep quality, and functional impairment in a community sample. J. Psychiatr. Res., 2009, 43: 926–933. 19269650 10.1016/j.jpsychires.2009.01.009

[jsr12282-bib-0036] Sharpless, B. and Barber, J. P. Lifetime prevalence rates of sleep paralysis: a systematic review. Sleep Med. Rev., 2011, 15: 311–315. 21571556 10.1016/j.smrv.2011.01.007 PMC3156892

[jsr12282-bib-0037] Sharpless, B. , McCarthy, K. S. , Cambless, D. L. , Milrod, B. L. , Khalsa, S. and Barber, J. P. Isolated sleep paralysis and fearful isolated sleep paralysis in outpatients with panic attacks. J. Clin. Psychol., 2010, 66: 1292–1306. 20715166 10.1002/jclp.20724 PMC3624974

[jsr12282-bib-0043] Sharpless, B. A. and Grom, J. L. Isolated sleep paralysis. Behav. Sleep Med., 2014, 14: 1–6. 10.1080/15402002.2014.963583 25315810

[jsr12282-bib-0038] Shi, S. , Hida, A. , McGuiness, O. P. , Wasserman, D. H. , Yamazaki, S. and Johnson, C. H. Circadian clock gene Bmal1 is not essential; functional replacement with its paralog, Bmal2. Curr. Biol., 2010, 20: 316–321. 20153195 10.1016/j.cub.2009.12.034 PMC2907674

[jsr12282-bib-0039] StataCorp . Stata Statistical Software: Release 9. Stata Corporation, College Station, TX, 2005.

[jsr12282-bib-0040] Szklo‐Coxe, M. , Young, T. , Finn, L. and Mignot, E. Depression: relationships to sleep paralysis and other sleep disturbances in a community sample. J. Sleep Res., 2007, 16: 297–312. 17716279 10.1111/j.1365-2869.2007.00600.x PMC2800990

[jsr12282-bib-0041] Takeuchi, T. , Fukuda, K. , Sasaki, Y. , Unugami, M. and Murphy, T. I. Factors related to the occurrence of isolated sleep paralysis elicited during a multi‐phasic sleep–wake schedule. Sleep, 2002, 25: 89–96. 11833865 10.1093/sleep/25.1.89

[jsr12282-bib-0042] Turner, S. , Armstrong, L. L. , Bradford, Y. *et al* Quality control procedures for genome wide association studies. Curr. Protoc. Hum. Genet., 2011, 68: 1.19.1–1.19.8. 10.1002/0471142905.hg0119s68 PMC3066182 21234875

